# Genetic analysis of the APC gene regions involved in attenuated APC phenotype in Israeli patients with early onset and familial colorectal cancer

**DOI:** 10.1054/bjoc.2001.1959

**Published:** 2001-08

**Authors:** A Figer, L Irmin, R Geva, D Flex, A Sulkes, E Friedman

**Affiliations:** The Institute of Oncology, Rabin Medical Center, Belinson Campus, Petach Tikvah; the Susanne Levy Gertner Oncogenetics Unit, The Danek Gertner Institute of Genetics, Chaim Sheba Medical Center, Tel-Hashomer, and the Sackler School of Medicine, Tel Aviv University, Ramat-Aviv, 52621, Israel

**Keywords:** germline mutations, attenuated APC, familial colorectal cancer, DGGE

## Abstract

The genetic basis for the majority of early onset or non-syndromic ‘familial’ colorectal cancer (CRC) is unknown. Attenuated APC phenotype is characterized by relatively few colonic polyps, early age at onset of colon cancer compared with the general population, and inactivating germline mutations within specific regions of the APC gene. We hypothesized that germline mutations within these APC gene regions, might contribute to early onset or familial CRC susceptibility. To test this notion, we analysed 85 Israeli patients with either early onset (< 50 years at diagnosis) or familial CRC for harbouring mutations within the relevant APC gene regions: exons 1–5, exon 9 and a region within exon 15 (spanning nucleotides c.3900 to c.4034; codons 1294 to 1338) using denaturing gradient gel electrophoresis (DGGE), and all of exon 15 employing protein truncation test (PTT). No inactivating, disease-associated mutations were detected in any patient. A novel polymorphism in intron 5 was detected in 16 individuals, 8 patients were carriers of the 11307K variant, a mutation prevalent among Jewish individuals with colorectal cancer, and 4 displayed the E1317Q variant. We conclude that in Israeli individuals with early onset or familial CRC, truncating mutations in the APC gene regions associated with attenuated APC phenotype probably contribute little to disease pathogenesis. © 2001 Cancer Research Campaign http://www.bjcancer.com


*British Journal of Cancer* (2001) **85**(4), 523–526
© 2001 Cancer Research Campaign
doi: 10.1054/ bjoc.2001.1959, available online at http://www.idealibrary.com on http://www.bjcancer.com

### Genetic analysis of the APC gene regions involved in attenuated APC phenotype in Israeli patients with early onset and familial colorectal cancer

**A Figer[1], L Irmin[2], R Geva[1], D Flex[1], A Sulkes[1] and E Friedman[2]**

[1]The Institute of Oncology, Rabin Medical Center, Belinson Campus, Petach Tikvah; and [2]the Susanne Levy Gertner Oncogenetics Unit, The Danek Gertner Institute of Genetics, Chaim Sheba Medical Center, Tel-Hashomer, 52621, and the Sackler School of Medicine, Tel Aviv University, Ramat-Aviv, Israel

**Summary** The genetic basis for the majority of early onset or non-syndromic 'familial' colorectal cancer (CRC) is unknown. Attenuated APC phenotype is characterized by relatively few colonic polyps, early age at onset of colon cancer compared with the general population, and inactivating germline mutations within specific regions of the APC gene. We hypothesized that germline mutations within these APC gene regions, might contribute to early onset or familial CRC susceptibility. To test this notion, we analysed 85 Israeli patients with either early onset (< 50 years at diagnosis) or familial CRC for harbouring mutations within the relevant APC gene regions: exons 1–5, exon 9 and a region within exon 15 (spanning nucleotides c.3900 to c.4034; codons 1294 to 1338) using denaturing gradient gel electrophoresis (DGGE), and all of exon 15 employing protein truncation test (PTT). No inactivating, disease-associated mutations were detected in any patient. A novel polymorphism in intron 5 was detected in 16 individuals, 8 patients were carriers of the 11307K variant, a mutation prevalent among Jewish individuals with colorectal cancer, and 4 displayed the E1317Q variant. We conclude that in Israeli individuals with early onset or familial CRC, truncating mutations in the APC gene regions associated with attenuated APC phenotype probably contribute little to disease pathogenesis.
© 2001 Cancer Research Campaign http://www.bjcancer.com

**Keywords**: germline mutations; attenuated APC; familial colorectal cancer, DGGE

Inactivating germline mutations within the adenomatosis polyposis coli (APC) gene underlie familial adenomatous polyposis (FAP), a dominantly inherited syndrome characterized by the development of hundreds to thousands of polyps in the colon and rectum beginning in the teen years, with the majority of patients developing colon cancer at about age 40. Additionally, variable extracolonic manifestations can also be detected in mutation carriers: gastric and duodenal polyps, osteomas, retinal lesions, and desmoid tumours (Groden et al, 1991; Leppert et al, 1990; Burt and Samowits 1988; Nishisho et al, 1991). Most inherited mutant alleles of the APC gene that are associated with the classical form of the FAP lead to truncation of the protein product, are scattered throughout the gene, and seem to be family specific (van der Luijt et al, 1997; Giarola et al, 1999; Wallis et al, 1999; Ficari et al, 2000). Several mutations are recurring in various ethnic groups, with some mutations attributed to a founder effect (Spirio et al, 1999) and other mutations, to a mutational hot spot (Beroud and Soussi, 1996). Notably, a 5 base pair deletion mutation (c.3927-c.3931 delAAAGA) known as codon 1309 termination mutation is the most common germline mutation detected (APC mutation database) and results in a severe form of FAP (Spirio et al, 1999; Wallis et al, 1999; Ficari et al, 2000).

A subset of FAP families have a less aggressive form of the disease, termed attenuated APC (AAPC), where the number of polyps in the colon is usually less than 100, with a later age at diagnosis of both polyposis and cancer than classical FAP (Spirio et al, 1993; Samowitz et al, 1995). In fact, some individuals who bear the AAPC alleles have as few as one polyp (Soravia et al, 1998). The regions within the APC gene that are associated with an attenuated phenotype include the 5´ end of the gene (coded by exons 1–5) (Spirio et al, 1993; Samowitz et al, 1995; Soravia et al, 1998), exon 9 (van der Luijt et al, 1995; Soravia et al, 1998; Young et al, 1998; Rozen et al, 1999), and the 3´ end of the large exon 15 (Scott et al, 1995; van der Luijt et al, 1996; Brensinger et al, 1998; Soravia et al, 1998; Matsubara et al, 2000). Surprisingly, even a cytogenetically visible interstitial 5q deletion that deletes the entire APC gene, intuitively predicted to result in a classical FAP phenotype, has been reported to result in an AAPC phenotype (Pilarski et al, 1999).

A novel missense mutation within the *APC* gene, I1307K, has been described initially in Jewish individuals of East European descent (Ashkenazi), both at risk for colorectal cancer (CRC), and also in the general, average risk, population of the same ethnic extraction (Laken et al, 1997, Woodage et al, 1998). Studies involving I1307K carriers have shown a moderate increase (up to 2 fold) in colon cancer risk (Laken et al, 1997; Rozen et al, 1999). This specific polymorphism was considered to be limited to Ashkenazi Jews (Laken et al, 1997; Prior et al, 1999), but it was recently found in Jewish individuals of non-Ashkenazi origin as well (Rozen et al, 1999; Patael et al, 1999).

About 10–15% of all CRC are attributed to the familial form of the disease, hallmarked by an earlier age at diagnosis (< 50 years) than the general population. Hereditary non-polyposis colon cancer (HNPCC) accounts for about 30–35% of familial cases, and

*Received 7 December 2000*
*Revised 28 March 2001*
*Accepted 30 May 2001*

*Correspondence to:* E Friedman classical form of familial adenomatous polyposis coli (FAP) accounts for only a minority of these familial cases. Yet, the majority of familial and early onset cases remain genetically unaccounted for. We hypothesized that APC germline mutations within gene regions associated with an attenuated phenotype, might contribute to 'familial' or early onset colorectal cancer. To test this notion, we analysed the relevant regions of the APC gene for germline mutations in a panel of Israeli patients with CRC who had either familial or early onset cancer, who were treated in a single medical centre in Israel.

### MATERIALS AND METHODS

### Patients

All analysed patients had clinical and pathological confirmation of CRC, and were being treated and followed up at the Oncology Institute at the Rabin Medical centre. The Institutional review board approved the study, and each participant signed a written informed consent. All clinical details were retrieved by a detailed questionnaire filled by the patients, and extraction of data from the medical records and pathology reports. Patients were designated as familial CRC if they had at least one additional first or two additional second-degree family members with CRC, regardless of age at diagnosis. Early ages at onset were patients in whom cancer was diagnosed under the age of 50 years. Patients who fulfilled the Amsterdam or Bethesda II criteria for HNPCC (OMIM # 114500) (OMIM database) were excluded from analysis. The control population included DNA stored at the Chaim Sheba Medical Center Genetics Institute, from women who were screened for carrier status of some of the common recessive diseases (e.g. Cystic fibrosis, Gaucher, Canavan).

### DNA extraction

Genomic DNA was prepared from anticoagulated venous blood samples using standard techniques, employing the Gentra Kit (Gentra Inc., Minneapolis, MN) and using the manufacturer's recommended protocol.

### PCR and DGGE analysis APC exons 1–5 and 9

Analysis of exons 1–5 and exon 9 of the APC gene was carried out using flanking intronic primers, and implementing the PCR protocols that were previously described (Olschwang et al, 1993). The resulting PCR products were subjected to denaturing gradient gel electrophoresis (DGGE) under the conditions described previously (Olschwang et al, 1993). All consistently abnormally migrating fragments (i.e. repeated abnormalities on three independent PCRs) were subject to sequence analysis using the Big Dye terminator kit (PE Biosystems, Foster City, CA), and using the ABI Prism 310 semiautomatic DNA sequencer (PE Biosystems).

### PCR and Protein Truncation Test (PTT) analysis of exon 15 of the APC gene

PTT analysis was used by adopting the protocol previously described (van der Luijt et al, 1997) and using the transcription translation kit by Promega (Madison, WI). The translated PCR products were analysed on polyacrylamide gels, vacuum-dried, and exposed to X-ray film for 24–48 h.

### Detection of the I1307K, 1309del5 and E1317Q variants by DGGE

Detection of these mutations included PCR amplification of the relevant genomic region contained in exon 15 (spanning nucleotide c.3900 to c.4034, codons 1294 to 1338) followed by DGGE analysis, using the protocol previously published by us (Patael et al, 1999). For analysis of the 11307K mutation, we also used a modified restriction analysis as detailed elsewhere (Shtoyerman-Chen et al, 2000). Briefly, the following primers were used to amplify the relevant region within exon 15 of the APC gene: forward primer (sense) 5′-GCA GAT TCT GCT AAT ACC CTG CAA ATA GCA TTAA-3′; reverse primer (antisense) 5′-CCT GAA GAA AAT TCA ACA GCT TTG TGC CTG-3′. PCR were performed using standard 10X PCR buffer, in a 50 μl reaction volume, using the M. J. Research PTC 100–60 thermocycler (M. J. Research Inc., Watertown, MA). Thirty cycles of amplification with the annealing temperature set at 55˚C for 1 min were employed. Following thermal cycling, 25–50% of the PCR products were digested with MvaI enzyme (New England Biolabs), for 3 h at 37˚C, and resolved on a 2.5% agarose gel with UV Transillumination. The mutant allele deletes the MvaI site. Abnormally migrating bands on DGGE that did not harbor the I1307K mutation were subjected to direct DNA sequencing, as detailed above.

### RESULTS

### Patients' and tumour characteristics

Overall, 85 patients were analysed: 48 men and 37 women. There were 50 patients of Ashkenazi origin, 22 non-Ashkenazis, 10 of mixed origin and 3 Israeli Arabs. The age at onset was 50± 13.8 years (median ± SD) range 24–84 years. A total of 50 patients (58.8%) had first-degree relatives with cancer: 37 (74%) had relatives with colorectal cancer, 5 (10%) with other gastrointestinal malignancies, 3 (6%) had relatives with breast/ovarian cancer and in 10 patients – other malignancies were noted in relatives. The majority of the patients (45/85–52.9%) had Dukes stage B, and 23 (27%) had Dukes stage C. Tumours were most commonly located at the rectosignmoid region ($n$ = 50–58.8%), in 18 (21.2%) the tumour was located in the right colon, 8 patients (9.4%) had CRC in the descending colon, and 8 others in the transverse colon. One patient had two distinct tumours: one in the caecum and the other in the rectosigmoid. Twenty-one patients (24.7%) had previous or ($n$ = 19) concurrent ($n$ = 2) colonic polyps, with six having more than one polyp.

### Mutation analyses of the relevant APC gene regions

No abnormal patterns suggestive of truncating mutations in PTT analysis of exon 15 were detected in any of the patients with any of the fragments (data not shown). DGGE analysis of exons 1– 4 and exon 9 did not display any abnormal migrating fragments suggestive of harboring sequence alterations. A common migration abnormality in the fragment containing exon 5 was detected: 14 samples showed a heterozygous pattern and two a mutant homozygous pattern. Sequence analysis revealed a T to a G change 32 bases from the splice junction of exon 5, in intron 5 (IVS5+32 T - > G) in all sequenced samples.

 © 2001 Cancer Research Campaign

### Analysis of the mutation cluster region within exon 15

Consistent migration abnormalities were detected in 12 patients in the mutation cluster region of exon 15. Of these, 8 (9.4% of all patients and 16% of the Ashkenazis) were I1307K mutation carriers, and the rest (*n* = 4) were E1317Q missense mutation carriers. Of the 21 patients with colonic polyps, two (9.5%) were I1307K mutation carriers and one (4.75%) carried the E1317Q missense mutation. No mutations in codon 1309 were detected (APC mutation database). Among the control population the I1307K mutation was detected in 8/148 (5.4%) Ashkenazi women, and the E1317Q mutation in two (2.7%) women. Naturally these tests were performed anonymously, no details as to the personal or family history of colonic polyps or cancer is available from these individuals.

### DISCUSSION

The rationale that led to analysis of the specific APC gene regions in the present study's patients is based on several observations. First, few if any mutations in any known CRC predisposition genes have been reported in the majority of early onset or non-syndromic 'familial' CRC patients. Second, the relatively mild phenotype that is associated with germline mutations within the APC gene in the regions analysed. The finding of significantly more FAP-associated extracolonic manifestations in seemingly sporadic CRC patients compared with controls (Dunlop et al, 1996), may also implicate germline mutations within the APC gene in the pathogenesis of non FAP CRC. Yet, in our group of patients, no truncating, disease-causing mutations were detected in the APC gene within the regions that are associated with attenuated APC phenotype.

One reason for not detecting mutations could be the lack of sensitivity of the mutation detection schemes used: DGGE and PTT. This seems unlikely, though, as both techniques have been applied to this specific gene, and have been shown to detect mutations within the analysed regions (van der Luijt et al, 1997). Moreover, in this study, a novel polymorphism and two known missense mutations were detected using DGGE. Alternatively, mutations in other regions of the gene that were not analysed in the present study (e.g., other exons, intronic or promotor sequences), or even major gene rearrangements not detected by PCR-based analyses could exist.

Another possible reason for not detecting existing mutations is patient selection criteria. Indeed, if more individuals with multiple colonic adenomas would be analysed, perhaps truncating mutations could be found, as these features are more prevalent in attenuated APC phenotype (Spirio et al, 1993; Samowitz et al, 1995). However, the criteria used for patient inclusion are well established and accepted. Moreover, confirmation as to the appropriate selection of patients is also provided by the rate of I1307K mutation carriers among the tested Ashkenazi individuals, a rate that is similar to that reported for familial CRC Jewish patients (Laken et al, 1997; Rozen et al, 1999).

Previous analysis of the APC gene in non-APC families has been previously reported in a few studies. Wallis and co-workers identified three missense mutations among 15 non-APC colorectal cancer in England (Wallis et al, 1999). In contrast, no mutations within the first 6 coding exons were detected among 40 familial or early onset British patients with CRC (Joyce et al, 1995). More recently, analysis of 79 patients with early onset and/or familial CRC whose tumors show no microsatellite instability, failed to detect truncating mutations in any of the APC exons (Boardman et al, 2001).

There were 12 individuals in this study who displayed one of two missense mutations within the APC gene: I1307K and E1317Q. The role of the I1307K mutation in predisposing to colon cancer is well established (Woodage et al, 1998; Rozen et al, 1999; Gryfe et al, 1999). Less certainty still exists as to the role of the E1317Q mutation in CRC predisposition. Frayling and co-workers (1998) initially detected this missense mutation in 4/164 (2.4%) patients with colon cancer in none of the controls, implicating an association of the E1317Q mutation in CRC pathogenesis. Moreover, this missense mutation was significantly more prevalent in individuals with multiple adenomas than in controls (Lamlum et al, 2000). However, analysis of a larger number of individuals revealed the mutation at a similar rate (about 0.5–0.6%) in both patients and controls (Popat et al, 2000). The data in the present study seem to be consistent with the notion that this variant represents a polymorphism, as it was detected in both patients and controls. However, a larger study encompassing more individuals, affected and controls, is certainly needed to assess the role, if any, that this mutation plays in CRC predisposition.

In conclusion, no inactivating, disease-causing mutations in the *APC* gene regions that are associated with an attenuated phenotype have been detected in Israeli patients with either familial or early onset colon cancer. The precise genes that do underlie this apparent inherited predisposition in these individuals remain elusive.

### ACKNOWLEDGEMENT

This study was funded in part by a generous donation from Mr Ami Yaar in loving memory of his wife, Ruti.

### REFERENCES

APC mutation database *http://perso.curie.fr/Thierry.Soussi/APC.html#Ancrage2*

Beroud C and Soussi T (1996) APC gene: database of germline and somatic mutations in human tumors and cell lines. *Nucleic Acids Res* **24**: 121–124

Boardman LA, Schmidt S, Lindor NM, Burgart LJ, Cunnigham LJ, Price-Troska TL, Show K, Ahlquist DA and Thibodeau SN (2001) A search for germline mutations in early onset or familial colorectal cancer with normal DNA mismatch repair APC. *Genes Chromosomes Cancer* **30**: 181–186

Brensinger JD, Laken SJ, Luce MC, Powell SM, Vance GH, Ahnen DJ, Petersen GM, Hamilton SR and Giardiello FM (1998) Variable phenotype of familial adenomatous polyposis in pedigrees with 3′ mutation in the APC gene. *Gut* **43**: 548–552

Burt and Samowit's (1998)

Dunlop MG, Farrington SM, Bubb VJ, Cunningham C, Wright M, Curtis LJ, Butt ZA, Wright E, Fleck BW, Redhead D, Mitchell R, Rainey JB, Macintyre IM, Carter DC and Wyllie AH (1996) Extracolonic features of familial adenomatous polyposis in patients with sporadic colorectal cancer. *Br J Cancer* **74**: 1789–1795

Ficari F, Cama A, Valanzano R, Curia MC, Palmirotta R, Aceto G, Esposito DL, Crognale S, Lombardi A, Messerini L, Mariani-Costantini R, Tonelli F and Battista P (2000) APC gene mutations and colorectal adenomatosis in familial adenomatous polyposis. *Br J Cancer* **82**: 348–353

Frayling IM, Beck NE, Ilyas M, Dove-Edwin I, Goodman P, Pack K, Bell JA, Williams CB, Hodgson SV, Thomas HJ, Talbot IC, Bodmer WF and Tomlinson IP (1998) The APC variants I1307K and E1317Q are associated with colorectal tumors, but not always with a family history. *Proc Natl Acad Sci USA* **95**: 10722–10727

Giarola M, Stagi L, Presciuttini S, Mondini P, Radice MT, Sala P, Pierotti MA, Bertario L and Radice P (1999) Screening for mutations of the APC gene in 66 Italian familial adenomatous polyposis patients: evidence for phenotypic

© 2001 Cancer Research Campaign differences in cases with and without identified mutation. *Hum Mutat* **13**: 116–123
Groden et al (1991)
Gryfe R, Di Nicola N, Lal G, Gallinger S and Redston M (1999) Inherited colorectal polyposis and cancer risk of the APC I1307K polymorphism. *Am J Hum Genet* **64**: 378–384
Joyce JA, Froggatt NJ, Davies R, Evans DG, Trembath R, Barton DE and Maher ER (1995) Molecular genetic analysis of exons 1 to 6 of the APC gene in non-polyposis familial colorectal cancer. *Clin Genet* **48**: 299–303
Laken et al (1997)
Lamlum H, Al Tassan N, Jaeger E, Frayling I, Sieber O, Reza FB, Eckert M, Rowan A, Barclay E, Atkin W, Williams C, Gilbert J, Cheadle J, Bell J, Houlston R, Bodmer W, Sampson J and Tomlinson I (2000) Germline APC variants in patients with multiple colorectal adenomas, with evidence for the particular importance of E1317Q. *Hum Mol Genet* **22**: 2215–2221
Lepper et al (1990)
Matsubara N, Isozaki H and Tanaka N (2000) The farthest 3′ distal end APC mutation identified in attenuated adenomatous polyposis coli with extracolonic manifestations. *Dis Colon Rectum* **43**: 720–721
Nishisho et al (1991)
Olschwang S, Laurent-Puig P, Groden J, White R and Thomas G (1993) Germ-line mutations in the first 14 exons of the adenomatous polyposis coli (APC) gene. *Am J Hum Genet* **52**: 273–279
OMIM database – http://www.ncbi.nlm.nih.gov/htbin-post/Omim/dispmim? 114500
Patael Y, Figer A, Gershoni-Baruch R, Papa MZ, Risel S, Chen R, Karasik A, Theodor L and Friedman E (1999) A common origin of the 11307K APC polymorphism in Ashkenazi and non-Ashkenazi Jews. *Eur J Hum Genet* **7**: 555–559
Pilarski RT, Brothman AR, Benn P and Shulman Rosengren S (1999) Attenuated familial adenomatous polyposis in a man with an interstitial deletion of chromosome arm 5q. *Am J Med Genet* **86**: 321–324
Popat S, Stone J, Coleman G, Marshall G, Peto J, Frayling I and Houlston (2000) Prevalence of the APC E1317Q variant in colorectal cancer patients. *Cancer Lett* **149**: 203–206
Prior et al (1999)
Rozen P, Samuel Z, Shomrat R and Legum C (1999) Notable intrafamilial phenotypic variability in a kindred with familial adenomatous polyposis and an APC mutation in exon 9. *Gut* **45**: 829–833
Samowitz WS, Thliveris A, Spirio LN and White R (1995) Alternatively spliced adenomatous polyposis coli (APC) gene transcripts that delete exons mutated in attenuated APC. *Cancer Res* **55**: 3732–3734
Scott RJ, van der Luijt R, Spycher M, Mary JL, Muller A, Hoppeler T, Haner M, Muller H, Martinoli S, Brazzola PL and Kahn PM (1995) Novel germline APC gene mutation in a large familial adenomatous polyposis kindred displaying variable phenotypes. *Gut* **36**: 731–736
Shtoyerman-Chen R, Friedman E, Figer A, Carmel M, Patael Y, Rath P, Fidder HH, Bar-Meir S and Theodor L (2000) The 11307K APC Polymorphism: Prevalence in non-Ashkenazi Jews and Evidence for a Founder Effect. *Genetic Testing* (In press)
Soravia C, Berk T, Madlensky L, Mitri A, Cheng H, Gallinger S, Cohen Z and Bapat B (1998) Genotype-phenotype correlations in attenuated adenomatous polyposis coli. *Am J Hum Genet* **62**: 1290–1301
Spirio L, Olschwang S, Groden J, Robertson M, Samowitz W, Joslyn G, Gelbert L, Thliveris A, Carlson M, Otterud B, Lynch H, Watson P, Lynch P, Laurent-Puig P, Burt R, Hughes JP, Thomas G, Leppert M and White R (1993) Alleles of the APC gene: an attenuated form of familial polyposis. *Cell* **75**: 951–957
Spirio L, Green J, Robertson J, Robertson M, Otterud B, Sheldon J, Howse E, Groden J, White R and Leppert M (1999) The identical 5′ splice-site acceptor mutation in five attenuated APC families from Newfoundland demonstrates a founder effect. *Hum Genet* **105**: 388–398
van der Luijt RB, Vasen HF, Tops CM, Breukel C, Fodde R and Meera Khan P (1995) APC mutation in the alternatively spliced region of exon 9 associated with late onset familial adenomatous polyposis. *Hum Genet* **96**: 705–710
van der Luijt RB, Meera Khan P, Vasen HF, Breukel C, Tops CM, Scott RJ and Fodde R (1996) Germline mutations in the 3′ part of APC exon 15 do not result in truncated proteins and are associated with attenuated adenomatous polyposis coli. *Hum Genet* **98**: 727–734
van der Luijt RB, Khan PM, Vasen HF, Tops CM, van Leeuwen-Cornelisse IS, Wijnen JT, van der Klift HM, Plug RJ, Griffioen G and Fodde R (1997) Molecular analysis of the APC gene in 105 Dutch kindreds with familial adenomatous polyposis: 67 germline mutations identified by DGGE, PTT, and southern analysis. *Hum Mutat* **9**: 7–16
Wallis YL, Morton DG, McKeown CM and Macdonald F (1999) Molecular analysis of the APC gene in 205 families: extended genotype-phenotype correlations in FAP and evidence for the role of APC amino acid changes in colorectal cancer predisposition. *J Med Genet* **36**: 14–20
Woodage T, King SM, Wacholder S, Hartge P, Struewing JP, McAdams M, Laken SJ, Tucker MA and Brody LC (1998) The APCI1307K allele and cancer risk in a community-based study of Ashkenazi Jews. *Nat Genet* **20**: 62–65
Young J, Simms LA, Tarish J, Buttenshaw R, Knight N, Anderson GJ, Bell A and Leggett B (1998) A family with attenuated familial adenomatous polyposis due to a mutation in the alternatively spliced region of APC exon 9. *Hum Mutat* **11**: 450–455

 *© 2001 Cancer Research Campaign*
